# COP9 signalosome subunit 5A affects phenylpropanoid metabolism, trichome formation and transcription of key genes of a regulatory tri-protein complex in Arabidopsis

**DOI:** 10.1186/s12870-018-1347-9

**Published:** 2018-06-25

**Authors:** Shu Wei, Xiang Li, Margaret Y. Gruber, Biruk A. Feyissa, Lisa Amyot, Abdelali Hannoufa

**Affiliations:** 1 State Key Laboratory of Tea Plant Biology and Utilization, Anhui Agricultural University, Hefei, Anhui China; 2 Agriculture and Agri-Food Canada, Saskatoon Research Center, Saskatoon, SK Canada; 3 Department of Biochemistry and Biomedical Sciences, Faculty of Health Sciences, McMaster University, Hamilton, ON Canada; 4 Agriculture and Agri-Food Canada and Department of Biology, University of Western Ontario, London, ON Canada

**Keywords:** CSN5a, Arabidopsis, COP9 signalosome, Anthocyanin, Trichomes, MYB75

## Abstract

**Background:**

Trichomes and phenylpropanoid-derived phenolics are structural and chemical protection against many adverse conditions. Their production is regulated by a network that includes a TTG1/bHLH/MYB tri-protein complex in Arabidopsis. *CSN5a*, encoding COP9 signalosome subunit 5a, has also been implicated in trichome and anthocyanin production; however, the regulatory roles of CSN5a in the processes through interaction with the tri-protein complex has yet to be investigated.

**Results:**

In this study, a new *csn5a* mutant, *sk372*, was recovered based on its altered morphological and chemical phenotypes compared to wild-type control. Mutant characterization was conducted with an emphasis on trichome and phenylpropanoid production and possible involvement of the tri-protein complex using metabolite and gene transcription profiling and scanning electron microscopy. Seed metabolite analysis revealed that defective CSN5a led to an enhanced production of many compounds in addition to anthocyanin, most notably phenylpropanoids and carotenoids as well as a glycoside of zeatin. Consistent changes in carotenoids and anthocyanin were also found in the *sk372* leaves. In addition, 370 genes were differentially expressed in 10-day old seedlings of *sk372* compared to its wild type control. Real-time transcript quantitative analysis showed that in *sk372*, *GL2* and tri-protein complex gene *TT2* was significantly suppressed (*p* < 0.05) while complex genes *EGL3* and *GL3* slightly decreased (*p* > 0.05). Complex genes *MYB75, GL1* and flavonoid biosynthetic genes TT3 and TT18 in *sk372* were all significantly enhanced. Overexpression of *GL3* driven by cauliflower mosaic virus 35S promotor increased the number of single pointed trichomes only, no other phenotypic recovery in *sk372*.

**Conclusions:**

Our results indicated clearly that COP9 signalosome subunit CSN5a affects trichome production and the metabolism of a wide range of phenylpropanoid and carotenoid compounds. Enhanced anthocyanin accumulation and reduced trichome production were related to the enhanced *MYB75* and suppressed *GL2* and some other differentially expressed genes associated with the TTG1/bHLH/MYB complexes.

**Electronic supplementary material:**

The online version of this article (10.1186/s12870-018-1347-9) contains supplementary material, which is available to authorized users.

## Background

Plants have developed sophisticated structural and chemical mechanisms to resist biotic and abiotic stresses for improving their survival and reproductive fitness. Trichomes, which are protruding epidermal structures on aerial tissues, can protect plants from ultraviolet (UV) light, insect damage, and excess transpiration. Many trichome-borne phytochemicals are involved in resistance against pathogens or in the attraction for pollinators [1, 2]. Phenylpropanoid-derived phenolics are a large class of plant secondary metabolites actively involved in chemical defenses against herbivores, microbial pathogens, UV-B, and free radicals [3, 4]. Phenylpropanoid metabolism begins with the synthesis of activated hydroxycinnamic acid (*p*-coumaroyl-CoA) and the downstream reactions are comprised of the lignin, flavonoid, and other species-specific pathways [5, 6]. In the lignin pathway, monolignols *p*-coumaryl, coniferyl, and sinapyl alcohols, respectively, produce H-, G-, and S-type lignins. In the flavonoid pathway, dihydroflavonols are produced from *p*-coumaroyl-CoA by the actions of early biosynthetic genes (EBGs) such as *CHALCONE SYNTHASE* and *FLAVANONE 3-HYDROXYLASE*. The downstream flavonoid pathway produces catechins, anthocyanins, proanthocyanidins, and other compounds including their glycosides by late biosynthetic genes (LBGs), such as *DIHYDROFLAVONOL 4-REDUCTASE* (*DFR*, *TT3*), *LEUKOANTHOCYANIDIN DIOXYGENASE* (*LDOX*, *TT18*) and *ANTHOCYANIDIN SYNTHASE* [6].

The molecular mechanism of trichome formation has been characterized extensively in Arabidopsis*.* Recent studies reveal that there are over 70 genes involved in trichome initiation and development [7, 8]. Among them, a few primary genes encode proteins that form di- /tri- protein complexes to regulate the initiation of trichome development [9]. Tri-protein complexes are composed of a R2R3 activator MYB GLABROUS1 (GL1), a bHLH protein GLABROUS3 (GL3) and/or ENHANCER OF GLABRA3 (EGL3) and a WD40 protein TRANSPARENT TESTA GLABRA1 (TTG1), while di-protein complexes do not contain TTG1. These MBW complexes regulate the initiation and patterning of trichomes and several inhibitory R3 MYB proteins such as CAPRICE (CPC) can compete with GL1 to form inactive complexes to inhibit trichome initiation [10]. Similar tri-protein complexes that contain a MYB protein PRODUCTION OF ANTHOCYANIN PIGMENT 1 (PAP1) or TRANSPARENT TESTA 2 (TT2) and TTG1 and TRANSPARENT TESTA 8 (TT8)/GL3 can enhance anthocyanin biosynthesis in Arabidopsis [11, 12] by inducing several late flavonoid biosynthetic genes (LBGs) such as TT3 and TT18 [13]. More recently, transcription of GLABRA2 (GL2) (a homeo-domain transcription factor) was found to be activated by some MBW complexes for trichome production in epidermal cells, which in turn directly represses the expression of MYB75 and TT8, consequently resulting in anthocyanin biosynthesis inhibition in Arabidopsis [14]. These findings clearly indicate anthocyanin production and trichome formation are affected by MBW complexes in Arabidopsis, depending on complex organization [5, 11, 15, 16]. Recently, SQUAMOSA PROMOTER BINDING PROTEIN-LIKE 9 (SPL9), a target of miR156, has been shown to disrupt the formation of the MBW complex via interacting competitively with R2R3-MYBs MYB75 to repress anthocyanin accumulation, and SPL9 also inhibits trichome initiation by activating some R3-MYB proteins to compete with GL1 for GL3/EGL3 binding [17].

In the loss-function mutants of the COP9 signalosome (CSN) subunit 5a and many other subunits, enhanced anthocyanin production and reduced trichome formation [18–21], as well as constitutive photomorphogenic phenotype and altered plant responses to some biotic stresses [22, 23], have also been documented. CSN complex consists of structurally interdependent eight subunits (CSN1-CSN8) and is a regulator of cullin–RING ubiquitin E3 ligases (CRLs) [24]. CSN regulates CUL1 ligases (commonly known as SCF complexes), which are involved in auxin signalling [25], flower development [26], jasmonate signalling [22] and gibberellic acid signalling [27] and many other pathways [21, 28, 29]. Therefore, CSN functions as a protease that cleaves RUB1/NEDD8 (both are ubiquitin-like protein covalently attached to CULLINs) in these E3 ligases [22, 24, 30, 31]. This catalytic centre is located in JAMM/MPN motif in CSN5 [20, 32–34]. Recent studies reveal that the function of SCF^COI1^, which is regulated by CSN [35], is involved in repression of anthocyanin accumulation and trichome initiation through the interactions between SCF^COI1^ substrate Jasmonate ZIM-domain (JAZ) proteins and MBW complex components (MYB75, GL1, GL3, EGL3, and TT8) [36]. However, SCF ^COI1^ regulation tends to inhibit both anthocyanin accumulation and trichome initiation simultaneously, which is different from the differential changes in anthocyanin and trichome production found in the null mutants of CSN5a [18, 19].

For the purpose to get a deep insight into CSN5a-induced alterations in phenylpropanoid metabolic pathways and trichome production and possible involvement of the TTG1/bHLH/MYB protein complex, characterization of a new Arabidopsis *csn5a* mutant *sk372* isolated from the Saskatoon Arabidopsis T-DNA population was conducted. Defective *CSN5a* was found to be responsible for enhanced production of numerous phenylpropanoids (including anthocyanins) and carotenoids in this mutant, as well as significantly reduced trichome density and distorted trichome morphology. These phenotypic alterations were closely correlated with enhanced MYB 75 expression and suppressed expression of *GL3* and *GL2*.

## Methods

### Chemicals

Authentic chemical standards β-carotene, lutein, β-cryptoxanthin, zeaxanthin, and violaxanthin were purchased from CaroteNature (www.carotenature.com). Butylated hydroxytoluene (BHT) and ampicillin were purchased from Sigma-Aldrich (www.sigmaaldrich.com/united-states.html) and corticosterone from Sigma Canada (www.sigmacanada.ca). All solvents used in this study were HPLC grade.

### Plant material and growth condition

*Arabidopsis thaliana* ecotype Columbia (*Col-4*) from the Arabidopsis Biological Resource Centre (www.arabidopsis.org) was used to develop the SRC (Saskatoon Research Centre) activation-tagged population [37]. Arabidopsis seeds were cold-treated at 4 °C for 2–4 days and then allowed to germinate and grow in soil in a greenhouse at 20 °C or on ½-strength Murashige and Skoog (MS) medium (Sigma, www.sigmaaldrich.com/canada-english.html) containing 0.8% agar and 4% sucrose in a 16 h light and 8 h dark regime. For epigenetic studies, the media was supplemented with 500 ng/mL of the histone deacetylation inhibitor Trichostatin A (TSA) (Sigma T8552) and Arabidopsis seedlings were germinated and grown in the above TSA media for 8 days according to Xu et al. [38].

### Molecular characterization of *sk372*

*sk372* was selected by screening T_3_ seeds of the SRC activation-tagged Arabidopsis population by visual examination for pale yellow leaves. The flanking sequences of Arabidopsis genome surrounding the T-DNA insert were isolated and sequenced using the gene-specific primer gwLB2 (Additional file 1: Table S1) and the Universal Genome Walker 2.0 Kit (www.clontech.com) according to the manufacturer’s instruction. The obtained sequencing results were analysed using BLASTN against the NCBI non-redundant database to identify the site of T-DNA insertion into the Arabidopsis genome. Southern blot analysis was conducted using 10 μg gDNA isolated from *sk372* seedlings and overnight digestion with *Hind*III or *Eco* RI by following a standard protocol. The T-DNA hybridization probe from pSKI015 and used to analyse the SRC activation-tagged Arabidopsis population was prepared as previously reported [39].

Complementation of *sk372* was performed with *CSN5a* and *GL3*. Full length native *CSN5a* coding sequence was amplified from WT seedlings with primer pairs P1 + P2 using high fidelity DNA polymerase *Pfu Phusion* (www.neb.com) (Additional file 1: Table S1). The amplified fragments were sequenced and cloned into *Xba* I and *Sma* I sites of the binary vector pBI121 under the control of the CMV*35S* promoter (35S). Plant expression vector containing 35S: *GL3* cassette constructed previously [40] was used for mutant trichome complementation. The constructs were first introduced into *Agrobacteria tumefaciens* strain GV3101 and then introduced into Arabidopsis by floral dipping [41]. Transformation-positive T_1_ transgenic plants were detected on ½-strength MS media using 50 μg/L kanamycin.

For analyses of mutant heterology and structure of the defective gene in *sk372*, total RNA was extracted from 14-d seedlings using a commercial RNAEasy mini kit (www.qiagen.com/us/search/rneasy-mini-kit/?akamai-feo=off). Genomic DNA contamination was minimized using on column DNasI digestion as kit manufacturer instructed. cDNA was then obtained using Superscript II reverse transcriptase (www.thermofisher.com/order/catalog/product/18064014). PCR analysis on the resulting cDNA was performed using the *Taq* DNA polymerase (www.thermofisher.com/order/catalog/product/18038042). Gene specific primers for *CSN5a* (Additional file 1: Table S1) were designed based on *CSN5a* mRNA sequence (AT1G22920). PCR conditions were set up as follows: 94 °C for 3 min, 25 and 30 cycles at 94 °C for 30 s, 55 °C for 30 s, and 72 °C for 1 min, followed at the end by 72 °C for 5 min.

### Analysis of specialized metabolites

Specialized metabolites in phenylpropanoid pathway were extracted from Arabidopsis seeds in triplicate as described by Lisec et al. [42] and Ayenew et al. [43] with some modifications. Briefly, 50 mg frozen seeds grinded to powder using RETCH-mill (www.retsch.com) and grinding stainless beads. One mL of pre-chilled extraction solution, methanol/chloroform/water (2.5/1/1 *v*/v/v), containing internal standards was added. Internal standards of ampicillin (1 mg/mL in water) (www.sigmaaldrich.com/canada-english.html) and corticosterone (1 mg/mL in methanol) (www.sigmaaldrich.com/canada-english.html) were used, because they are not Arabidopsis metabolites and detected abundance in the LC-MS/MS is simply from the use of standards and will normalize the machine irregularities and pipetting variations as previously reported [43–45]. The mixture was vortexed and ultra-sonicated for 10 min each. Following centrifugation at 14000RPM for 10 min (at 4 °C) the supernatant was collected and mixed with equal volumes of 300 μL water and chloroform. The mixtures were vortexed briefly and centrifuged at 14000 RPM for 5 min to collect the upper aqueous phase for LC-MS^2^ analysis. LC-MS^2^ analysis was performed using an Agilent 1290 Infinity LC system coupled with a Thermo Q-Exactive Quadrupole-Orbitrap mass spectrometer. Samples were separated with Agilent Eclipse Plus C18 ZORBAX Rapid Resolution High Definition (RRHD) high resolution 1.8 μm particle 2.1 i.d. X 50 mm column as previously reported [45]. Detection of metabolites are done in positive and negative electrospray ionization mode (ESI) depending on the metabolites while the internal standards can be detected well in those conditions and to be used for normalization. Metabolites were identified based on mass to charge ratio (*m/z*), retention time and fragmentation pattern in comparison to commercial standards, ChemSpider and ReSpect phytochemical databases [43–45].

Carotenoids extracted in triplicate batches from Arabidopsis seed or 14-d seedling leaves were analysed as described by Kormendi et al. [46] with minor modifications. Briefly, 50 mg frozen seeds or leaf tissues were ground using RETCH-mill (Retsch Gmbh, 42,787 Haan, Germany) and extracted twice using 750 μL of extraction solvent (2:1:1 hexanes:acetone:ethanol). Following each extraction, samples were mixed in orbital shaker for 5 min at 1000RPM followed by centrifugation at full speed. Supernatants from both extraction were pooled and dried using a stream of nitrogen gas. The samples were resuspended in 1 mL solvent mixture of 5:4:1 acetonitrile:dichloromethane:methanol containing 0.5% (*w*/*v*) butylated hydroxytoluene (BHT). Finally, extracts were filtered by 0.2 μm nylon syringe filter and placed in a 2 mL amber HPLC vials. HPLC/UV analysis was conducted using a Hewlett Packard Agilent 1100 chromatograph, a G-7120 diode array detector coupled with Zorbax C_18_ column (150 × 4.6 mm, 5 μm ID) (www.agilent.com) and HP Chemstation ver. 8.01 software. Sample injection volume of 20 μL was used for analysis. Carotenoid identification and quantification were conducted using authentic standard compounds and calibration curves established with these standards as described before [47].

For chlorophyll measurements, 14-day-old seedlings of *Col-0* and *sk372* plants grown on ½ MS plus 4% sucrose [48] was extracted in 80% (*v*/v) acetone saturated with CaCO_3_ in the dark at 20 °C. Anthocyanins were extracted in 1% (v/v) HCl in methanol. These pigments were assayed in the cleared supernatants after 5 min centrifugation at 10,000 g.The chlorophyll and anthocyanins were quantified according to Mancinelli and Walsh [49].

### Microarray analysis

Total RNAs of Arabidopsis *Col-0* and *sk372* lines were extracted from seedlings grown on 1/2 MS agar plates containing 4% sucrose for 10 d and used in the preparation of Cy5- and Cy3-labelled cDNA probes, respectively. An Arabidopsis 26 K cDNA microarray (Qiagen-Operon Arabidopsis Genome Array Ready Oligo Set Version 1.0, cals.arizona.edu/microarray) was hybridized with Cy5- and Cy3-labelled probe pairs from the *Col-0* and *sk372* samples, respectively. Probe labelling, hybridization and washing were conducted using a CyScribe Post-Labelling Kit according to the manufacture’s direction (www.selectscience.net/suppliers/amersham-biosciences-corp/?compID=2504). Hybridized slides were scanned on a Gene Pix 4000B scanner (www.moleculardevices.com). The intensity (I) of each spot at λ_546nm_ and λ_647nm_was transformed into a ratio I_546_:I_647_ with the use of Array-Pro Analyzer 4.0 software (http://www.winsite.com/array/array+pro+analyzer/). Microarray data were initially imported into BASE 1.2.16 and Gene-Spring 6.1 (www.whitelabs.org/Lab%20Protocols/Genespring/genespring%2061.pdf) using log_2_ scale was used to select spots with intensity values > 50 in at least half of the samples (slides) for further analysis according to Zhao et al. [50]. Data normalization using the per-spot and per-chip Lowess normalization procedure with a smooth factor of 20 and a one-way ANOVA with a parametric test were performed with the value of false discovery rate (FDR) < 0.0001. A list of genes for which the expression varied by more than 2.0-fold was generated. Gene Ontology (GO) analysis was conducted using the TopGO package [51]. The GO enrichment analysis was performed by selecting both Fisher’s exact test and a Kolmogorov-Smirnov like test with *p*-values < 0.05 and the biological processes of affected GO terms were visualized according to Alexa et al. [51]

### Gene transcript analysis using quantitative real time PCR (qPCR)

RNA aliquots from the microarray experiment were also used in quantitative real-time PCR reactions with SuperScript III First-Strand Synthesis SuperMix and a qRT-PCR kit (www.thermofisher.com/order/catalog/product/11752050) according to the manufacturer’s instructions. DNase I was used for on column DNA digestion to minimize genomic DNA contamination. Real-time PCR analysis was carried out using Platinum SYBR Green qPCR SuperMix-UDG kit (www.thermofisher.com/order/catalog/product/11733046?SID=srch-srp-11733046) on an ABI PRISM StepOnePlus Real-Time PCR System (www.thermofisher.com/order/catalog/product/4376600), following the manufacturer’s instructions. Genes *ACT2* (AT3G18780) and *Ef-1α* (AT1G18070) were chosen as endogenous reference genes. For each pair of primers (Additional file 1: Table S1), gel electrophoresis and melting curve analyses were performed to ensure that only a single PCR amplicon of the expected length and melting temperature was generated. All primers used in Real-time RT-PCR analysis are listed in Additional file 2 Table S3. Each sample was assayed in triplicate and data were analysed using the StepOne Software v2.0 (www.advanceduninstaller.com/StepOne-Software-v2_1-bb54a0998d999a0cecd812484ca1bf64-application.htm). The level of each mRNA was calculated using the mean threshold cycle (Ct) value and normalized to that of the reference genes. All results were shown as means of at least three biological replicates with corresponding standard deviations (SD).

### Statistical analysis

Compositional data were expressed as mean ± standard error. All measurements were made from triplicate experiments and the data was analysed by ANOVA for significance (LSD at *P* < 0.05) using SAS 9.0 (SAS Institute, Inc. Cary, NC, USA, www.sas.com/en_us/home.html). For microarray analysis, background-corrected, log ratios of intensity were scaled to have similar distribution across and consistency among arrays. The ANOVA analysis was performed to detect differentially expressed genes using the normalized data [52].

## Results

### Phenotypic alterations of the *sk372* mutant

**Fig. 1:**
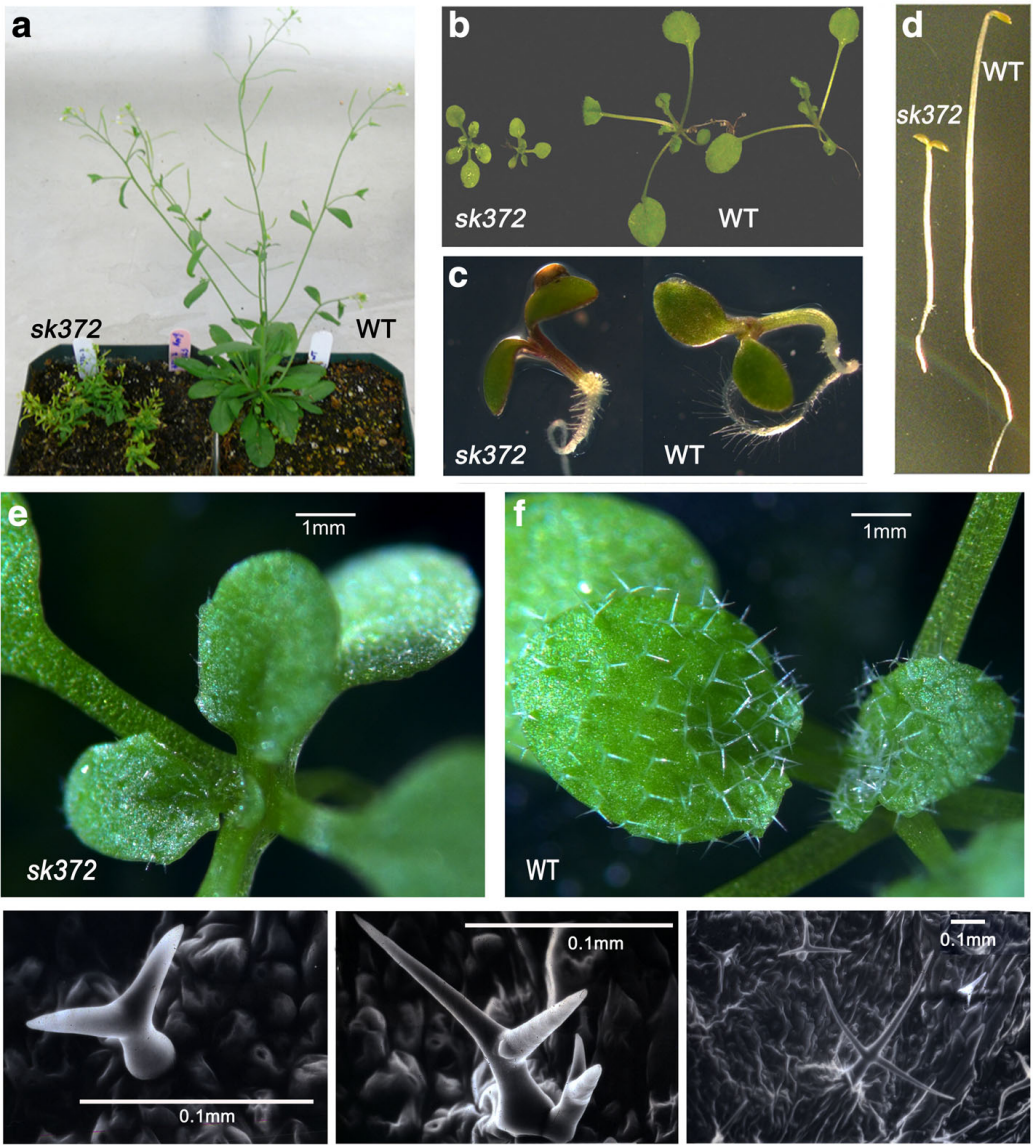
Morphological alterations of the *sk372* mutant compared to wild-type plants. **a** 45-day old plants; **b** 17-day old plants; **c** 4-day old plants in MS media containing 4% sucrose; **d** 4-day old plants grown in the dark; **e** leaf trichomes of 7-day old *sk372* plants; **f** leaf trichomes of 7-day old WT plants; **g, h** leaf trichomes of 7-day old *sk372* plants under scanning electronic microscopy; **i** leaf trichomes of 7-day old WT plants under scanning electronic microscopy

The *sk372* mutant was isolated from an *Arabidopsis thaliana* Columbia (Col-4) T-DNA activation tagged mutant population [37]. Compared to wild-type (WT) Arabidopsis, *sk372* exhibited several morphological alterations. Plants grown in different conditions were employed for morphological characterization throughout their life cycle. Plant growth was significantly inhibited at mature and rosette stages and mutant leaves were slightly yellowish (Fig. 1a and b); however, the mutant was capable of producing fertile seeds. Mutant seedlings grown in MS medium with addition of 4% sucrose had visibly purple stems and cotyledon edges (Fig. 1c). In darkness, WT seedlings showed typical phenotypes of skotomorphogenesis, such as elongated stems, undifferentiated chloroplasts, unexpanded cotyledons, and a germination hook (Fig. 1d), whereas the mutant seedling displayed some phenotypes of photomorphogenesis such as stunted but straight stems without a germination hook and expanded but etiolated cotyledons (Fig. 1d). Moreover, the trichome density observed on the adaxial surface of the fifth true leaf was 3.6 ± 0.4 per mm^2^, while the trichome density in WT was 6.5 ± 0.4 (Fig. 1e and f). Abnormal trichome size and shapes with fewer trichome branches were noted in the *sk372* mutant (Fig. 1g and 1e) compared to WT trichomes (Fig. 1i).

**Fig. 2:**
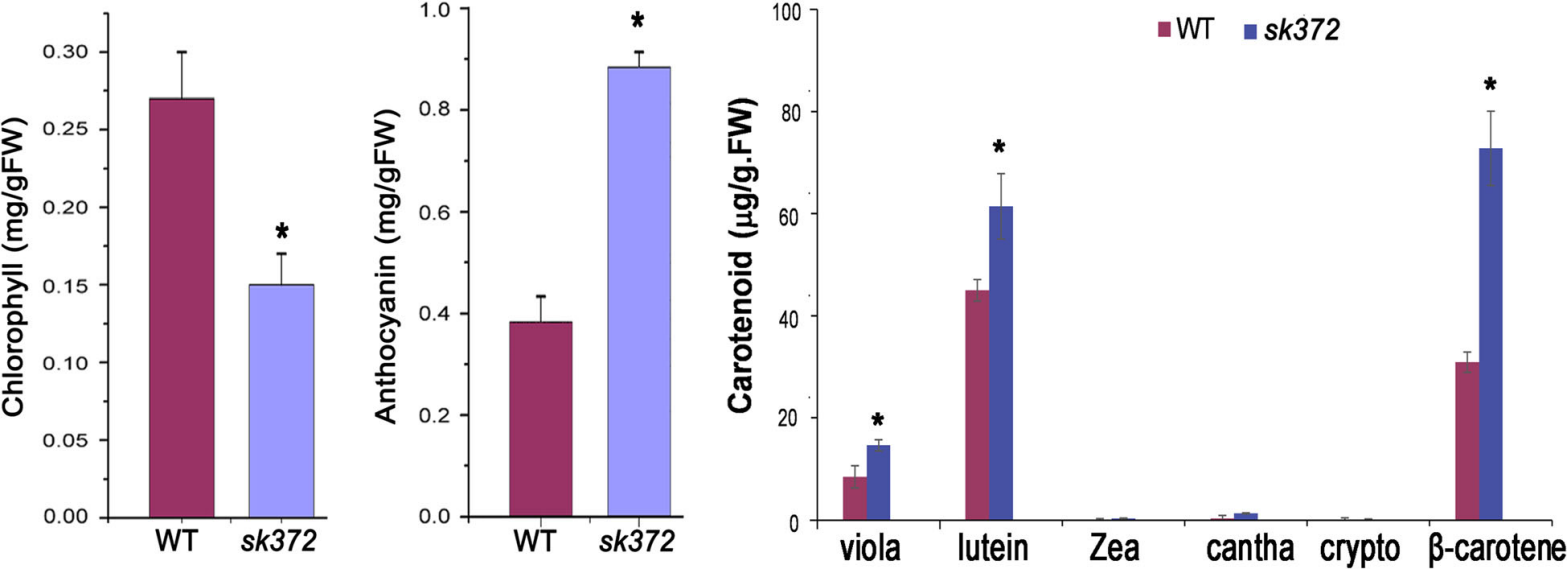
Leaf pigment abundances in 14-day old plants of *sk372* and WT. Significant difference (*) (*p* < 0.05) in the pigment abundance (total abundance for carotenoids) was found between *sk372* and WT. All measurements were made from triplicate experiments and the data was analysed by ANOVA for significance (LSD at *P* < 0.05) using SAS 9.0. viola, violaxanthin; zea, zeaxanthin; cantha, canthaxanthin; crypto, cryptoxanthin

For a better understanding of the observed leaf colour changes in *sk372*, leaf pigment analysis was conducted. Our data revealed that *sk372* leaves had a substantially lower level of chlorophyll but higher levels of total carotenoids and anthocyanin than WT (*p* < 0.05) (Fig. 2). In *sk372*, the levels of violaxanthin, lutein, and β-carotene were all significantly higher than their corresponding levels in WT (p < 0.05). The mutant contained slightly higher levels of minor carotenoids zeaxanthin (0.37 μg/g.FW) and canthaxanthin (1.36 μg/g.FW) compared to their respective controls (0.14 μg/g.FW and 0.39 μg/g.FW) (*p* > 0.05).

### Gene expression alteration in *sk372*

Additional investigations were undertaken to better understand the molecular mechanisms that lead to the above metabolic alterations in the *sk372* mutant. Microarray analysis using 10-day old seedlings, which were grown in MS media with 4% sucrose and exhibited visible colour changes, suggested that 370 genes were differentially expressed (> 2-fold) between WT and *sk372* seedlings. A total of 49 GO terms for biological processes were significantly affected, including responses to stimulus, anatomical structure development, and chromosome condensation and organization (Additional file 3: Figure S1). Out of 370 differentially expressed genes (DEGs), 115 suppressed genes coded for proteins that included those related to light reactions, such as the photosystem II 5 KD protein (At1g51400), a putative photosystem II type I chlorophyll a/b binding protein (At1g29910), and the light-harvesting chlorophyll a/b binding protein (At5g54270) (Additional file 4: Table S2). Suppressed genes that were related to sugar metabolism, cell wall and some amino acid biosynthesis were also found. In contrast, 255 genes were enhanced in *sk372* seedlings, including genes involved in sugar metabolism as well a gene in the flavonoid pathway (Additional file 2: Table S3). It was noted that in *sk372,* the enhanced expression of the genes mediating ubiquitin ligase activity was found 2.3- ~ 6.1-fold higher for five RIGN finger protein genes (At1g66040, At5g63780, At4g08590, At4g01270, and At2g42360) and 2.2- ~ 6.4-fold higher for seven F-box protein genes (At4g05470, At5g07610, At5g44980, At5g56810, At3g13680, At1g56240, and At3g59210) compared to WT counterparts. Expression of a putative geranyl diphosphate synthase gene (*GPPS*) (At1g78510), functioning upstream of carotenoid biosynthesis, was enhanced in *sk372*. Moreover, transcription of the UDP-glycosyltransferase gene UGT73C1(At2g36750), specifying glycosylation of cytokinins trans-zeatin and dihydrozeatin [53], was enhanced 7-fold in *sk372* seedlings. However, no DEGs (with ≥2-fold change in gene expression) related to trichome development were identified from *sk372* in the microarray analysis.

**Fig. 3:**
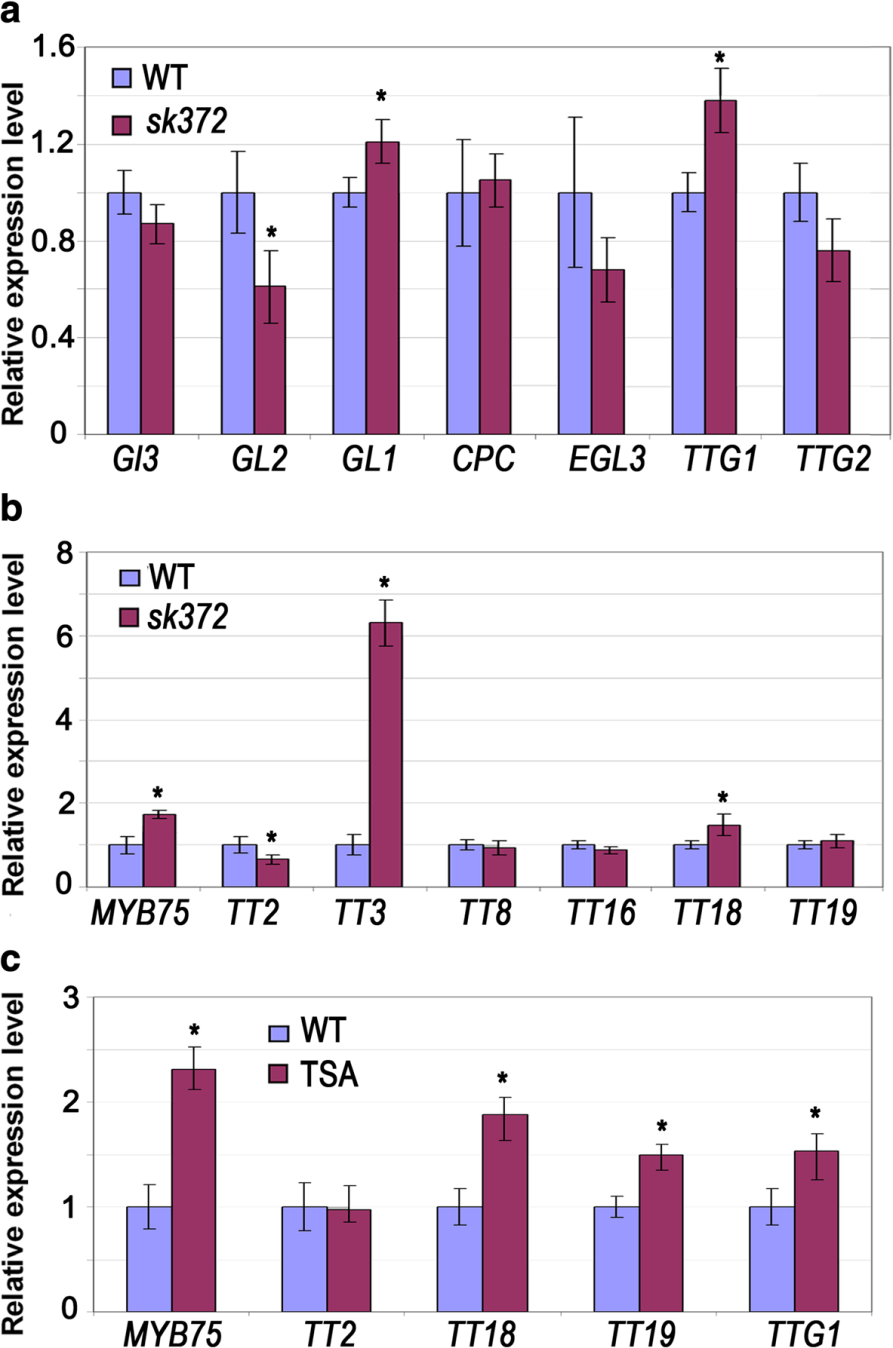
Transcript levels of genes related to trichome production, flavonoid metabolism, and tri-protein complexes in *sk372* mutant relative to WT control. Values for WT were set to an arbitrary number of 1. **a** Trichome production. **b** Flavonoid metabolism. **c** MBW tri-protein complex genes in Arabidopsis seedlings which were germinated and grown for 8 days in the MS media containing 500 ng/mL of the histone deacetylation inhibitor TSA

Since MBW (MYB-bHLH-WDR) complexes regulate flavonoid biosynthesis and trichome development [16, 54], we expected that some of the genes associated with these complexes and their regulatory pathways may be differentially expressed. Therefore, further quantitative PCR analyses were conducted to determine the expression levels of trichome development genes, the late stage genes of flavonoid pathways (downstream of dihydroflavonol biosynthesis), and the key genes in the MBW complexes in *sk372* and WT. Our data revealed that *sk372* exhibited lower transcript levels of the trichome-related homeodomain leucine zipper protein *GLABROUS2* (*GL2*) (*p* < 0.05)*,* but higher levels of *TTG1* and *GL1* (*p* < 0.05)*,* compared to WT seedlings (Fig. 3a). Moreover, the expression levels of *TT2* and the late stage flavonoid synthetic genes *TT3* and *TT18* were differentially expressed (*p* < 0.05) in the *sk372* mutant (Fig. 3b), with *TT3* and *TT18* being enhanced 6.4-fold and 1.3-fold, respectively, and *TT2* being slightly reduced. *MYB75* transcription level in *sk372* was also higher than in WT (*p* < 0.05).

Since GO term analysis had revealed that chromosome organization genes were affected and the transcript level of bromodomain protein BRD5 (At1g58025), potentially involved in histone acetylation [55] was reduced by 31-fold in *sk372* seedlings compared with WT plants the histone deacetylation inhibitor trichostatin A was applied to WT seedlings. This enabled us to examine the effects of histone deacetylation on the transcription of some anthocyanin genes. Our data indicated that expression of *MYB75* was enhanced more than 2-fold and that *TTG1*, *TT18* and *TT19* (*GLUTATHIONE S-TRANSFERASE*) were also enhanced (*p* < 0.05) with TSA application (Fig. 3c). Such an increase in the transcripts of all these genes except TT19 was also found in *sk372*, suggesting the possible involvement of epigenetic regulation of the mutant phenotype expression.

### Genetic mutation analysis of *sk372*

**Fig. 4:**
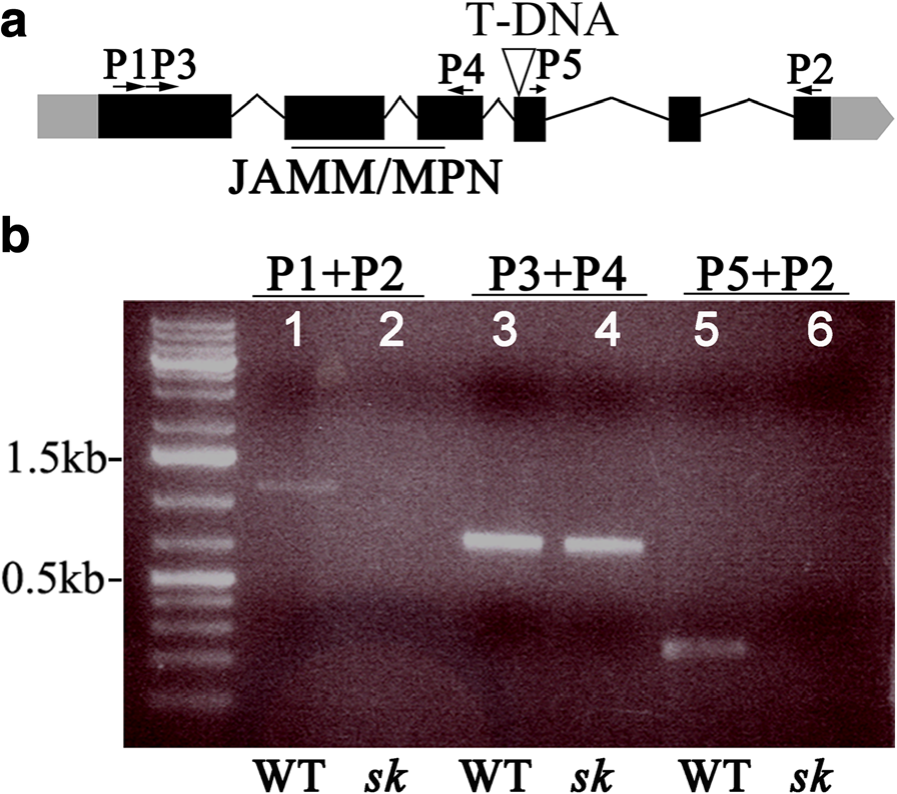
Molecular characterization of *sk372.* **a** Schematic representation of the *CSN5a* locus in *sk372* showing T-DNA insertion and primer locations. UTRs and exons are indicated by the grey and black boxes, respectively and introns are represented by lines. The CSN5a JAMM/MPN domain is underlined; **b** *CSN5a* truncation analysis using cDNA as a template. Lanes 1 and 2, full length coding sequence (938 bp) of *CSN5a* in WT and *sk372*, respectively; Lanes 3 and 4, amplified fragment from 162 bp to 886 bp of in WT and *sk372*; Lanes 5 and 6, amplified fragment starting from bp 1034 to 3′ end in WT and *sk372*

A genome-walker approach revealed that the T-DNA was inserted in the *COP9 SIGNALOSOME 5A* (*CSN5a*) (At1g22920) at the beginning of the fourth exon downstream from the JAMM/MPN motif (Fig. 4a), resulting in a truncated CSN5a, which contained the first 252 amino acid residuals including MPN domain and additional 17 residuals plus a stop codon (LHGSAMSMMVNMEEKKE*) due to the inserted T-DNA sequence, but lacking of 96 amino acid residuals at the C-terminus from the wild type CSN5a. Homozygosity of the T-DNA insertion in the CSN5a gene of *sk372* was confirmed by multiplex PCR using gene-specific primers spanning the full length cDNA and a primer specific to the T-DNA left border (Additional file 5: Figure S2). Southern blot analysis of the *sk372* mutant demonstrated a single copy insertion of the T-DNA in the mutant and homozygosity of the T-DNA insertion in the *CSN5a* gene of *sk372* was confirmed by multiplex PCR using gene-specific primers spanning the full-length cDNA and a primer specific to the T-DNA left border (Additional file 5: Figure S2). Reverse transcript-PCR analyses confirmed that the *sk372* mutant did not contain the full-length cDNA of *CSN5a*. Instead, a truncated *CSN5a* transcript containing the JAMM/MPN motif was detected in the *sk372* mutant (Fig. 4b).

### Complementation of *sk372* with *CSN5a*

**Fig. 5:**
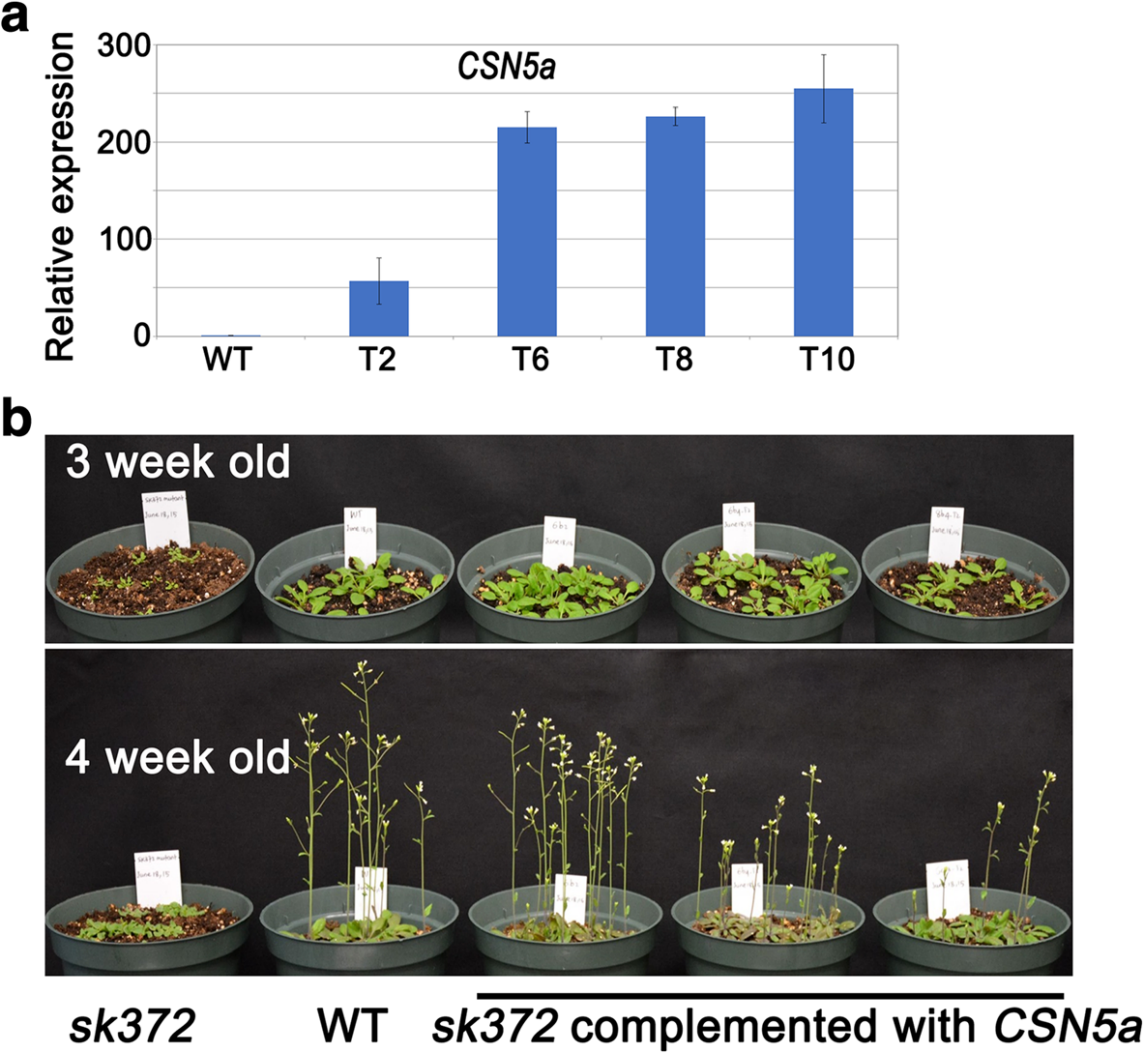
Genetic complementation of *sk372* with *CSN5a*. **a** Transgene (*CSN5a*) expression in the complemented *sk372*; **b** Restored phenotypes of the complemented *sk372* plants

The *sk372* mutant was complemented with the wild type coding sequence of *CSN5a* driven by the cauliflower mosaic virus 35S (CAMV35S) promoter and transgene expression was enhanced in some complemented T3 offspring (Fig. 5a). Plant morphology was restored in *sk372* complemented with the *CSN5a* transgene (Fig. 5b). Our data clearly indicated that disrupted CSN5a was responsible for the morphological alterations observed in *sk372*.

### Metabolic alteration and restoration in *sk372* and the complemented mutant

**Fig. 6:**
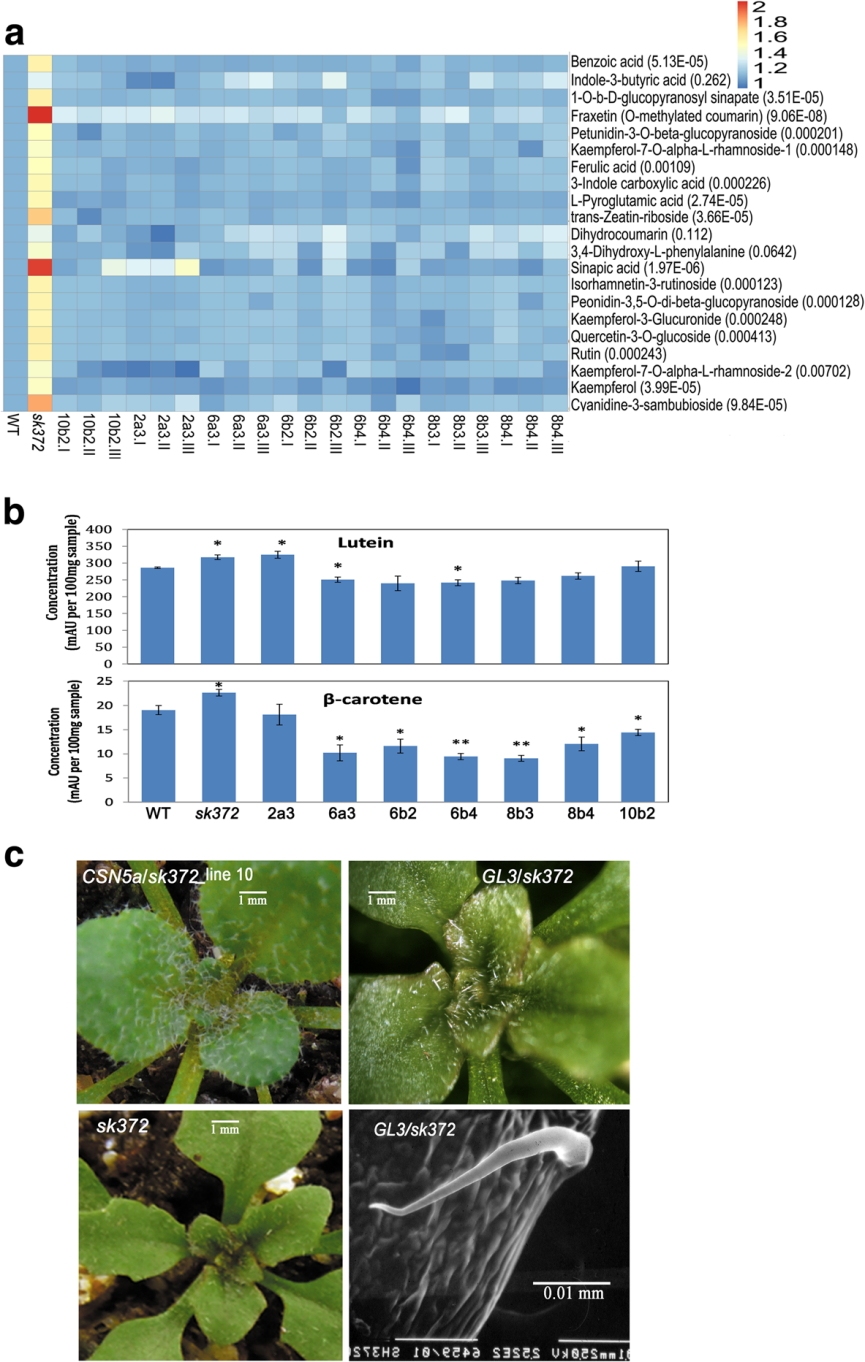
Alteration of metabolites and trichome development in *sk372* and their restoration in the CSN5a complementation lines. **a** Seed metabolites largely comprising phenylpropanoids and flavonoids. Numbers in the brackets after the compounds in the heatmap are the probabilities of relative abundance difference analysis; **b** Seed carotenoid levels, ‘*’ indicates significantly different from WT, p < 0.05; **c** Close-up examination of seedling trichomes in *sk372* complemented with *CSN5a* or *GL3*. WT, non transgenic plant of Col-0; *sk372*, T-DNA activation tagged mutant; 10b2I, -II, -III, three biological replicates of CSN5a complementation line 10b2 of T3 generation; 2a3, 6a3, 6b2, 6b4, and 8b3, and 8b4, CSN5a complementation lines of T3 generation. Columns with * and ** were significantly different from WT, p < 0.05 and *p* < 0.01

Leaf pigment analysis indicated that metabolic alterations occurred in leaves of the mutant. Seed metabolite analysis was also conducted for the purpose to verify whether leaf metabolic changes occurred similarly in seeds, the part of many crops often used as human food and with great significance in agricultural industries for the improvement of crop productivity and nutrition. This analysis might shed light on the potential to practically redirect metabolic pathways for the enforced carotenoids and flavonoids in crop seeds. In *sk372* seeds, many metabolites from the pathways of phenylpropanoids and flavonoids were enhanced substantially compared to WT seeds (Fig. 6a and b) (*p* < 0.05). Strikingly, the levels of 9 metabolites in the late stage of flavonoid biosynthesis increased, including kaempferol and kaempferol glycosides, quercetin glycoside, rutin, peonidin and petunidin (both *O*-methylated anthocyanidins), and isorhamnetin (*O*-methylated derivative of quercetin). Moreover, substantially higher levels of intermediate compounds in the lignin pathway, including ferulic acid, *O*-methylated coumarin, sinapic acid and its derivatives, were noted in *sk372* seeds than WT. Benzoic acid, which can be derived from the phenylpropanoid pathway [56], was also enhanced in *sk372*. seeds. Moreover, lutein and β-carotene, dominant carotenoid compounds in Arabidopsis, were enhanced somewhat compared to WT seeds (Fig. 6b). Interestingly, other compounds such as indole-3-carboxylic acid and zeatin-glycoside were also increased in the *sk372* mutant. All the alterations in relative abundance were fully or partially restored to varying degrees in the *sk372* lines and different *CSN5a* complementation lines as presented in the heatmap illustration (Fig. 6a). Our data revealed that disruption of CSN5a could lead to metabolic flux redirection in the pathways of seed phenylpropanoids, flavonoids, carotenoids, and even cytokinin glycosylation.

CSN5a overexpression in *sk372* was able to restore the entire WT trichome phenotype to the mutant transgenic lines (Fig. 6c). However, overexpression of *GL3* alone in the *sk372* background (as shown in Additional file 6: Figure S3) resulted in visually increased trichomes in the adaxial surface of the young leaves in *sk372* complemented lines compared to *sk3732* (Fig. 1e). The trichomes produced were largely single pointed (Fig. 6c), not tri-branched as in WT (Fig. 1f and i).

## Discussion

In this study, characterization of an Arabidopsis mutant *sk372* revealed that T-DNA disrupted CS*N5a* gene exhibited significant changes in several metabolic pathways, transcriptomic programming, trichome production, and plant morphology, although some similar changes have been documented in *csn5a* mutants previously [18–21]. In *sk372*, diverse metabolites including many phenylpropanoids, some carotenoids, and a glycoside of zeatin were found being enhanced while chlorophylls *a* and *b* were reduced and the altered expression of numerous genes including some primary genes of the tri-protein (MBW) complexes were revealed.

Microarray analysis combined with a follow-up expression validation using qRT-PCR is performed generally for the purpose to reveal possible differences in global gene transcription of tested samples. In this study, our microarray data suggested that in *sk372*, expression of *GPPS* (a carotenoid biosynthesis gene) and *UGT73C1* (a trans-zeatin glucotransferase gene) was elevated, which was correlated with the enhanced production of carotenoids and *trans*-zeatin riboside. Such a positive correlation is supported by functional studies on *GPPS*, a crucial gene upstream in carotenoid pathway by Kirby and Keasling [57] and UGT73C1 (At2g36750), that catalyses glycosylation of *trans*-zeatin and dihydrozeatin by Hou et al. [53]. Our data also indicated that F-box protein and RING finger protein genes were upregulated in *sk372*, suggesting that the expression of some genes encoding ligase mediators was also affected due to the defective CSN5a. This is not surprising because CSN5a is crucial for functional regulation of cullin-based ubiquitin E3 ligases [20, 33, 34] and defective CSN5a might consequently alter the expression of some E3 ligase mediators in *sk372*.

It was noted that our microarray analysis did not show any genes with altered transcription related to the phenylpropanoid pathway, carotenoid metabolism (except for *GPPS*), or trichome production between *sk372* and WT, even though the non-stringent DEG selection criteria of a 2-fold change was used. We speculated this was due to more subtle differences that were less than the cut-off value of two-fold. Moreover, quantitative PCR is generally considered more sensitive than microarray method [58, 59]. In fact, when we compared transcript levels of key MBW complex genes and some of their downstream targets quantified by qRT-PCR, we noticed that, with the exception of TT3, all were less than 2-fold difference between the WT control and *sk372* mutant. One explanation for the subtle differences could be due to the fact that RNA was extracted from whole seedlings and some of the genes of interest exhibit tissue-specific expression. For example, GL1 is expressed most strongly in developing trichomes [60], while GL3 transcripts accumulate in both root hairs and trichomes [61].

CSN-regulated anthocyanin accumulation and trichome initiation via SCF^COI1^ occurs through the interaction between the SCF^COI1^substrate JAZ and MBW complex components [36]. Moreover, miR156 enhances anthocyanin and trichome production by suppressing its target SPL9 which destabilizes MBW complexes [17]. However, in *sk372*, phenylpropanoids including anthocyanin were enhanced while trichome initiation was suppressed, which is different from the simultaneous enhancement or suppression of anthocyanin and trichome production as regulated by miR156-targeted SPL9 and CSN-affected SCF^COI1^. These inconsistent findings suggested that other mechanisms might be involved in *sk372*. Our qRT-PCR analysis revealed differential expression of several primary genes of the MBW complexes in the *sk372* mutant. *MYB75*, *TTG1* as well as flavonoid synthetic genes *TT3* and *TT18* were all enhanced in *sk372*. Consistently, many phenylpropanoids and their diverse derivatives were found being increased in *sk372*, in agreement with the finding that enhanced *MYB75* can lead to the significantly increased levels of similar phenylpropanoids such as hydroxycinnamic acid esters, and flavonoids, lignin, and anthocyanins that are responsible for the purple colour [62]. Differential expression of the MBW complex genes has been also reported earlier when the expression patterns of these genes were spatially compared in different organs in Arabidopsis [63]. These data together suggested that the MBW complexes, most likely with MYB75 outcompeted over GL1 for bHLH binding in *sk372*, resulted in the enhanced LBG genes *TT3* and *TT18* as reported before [13], in favour of the phenylpropanoid biosynthesis.

Moreover, the expression of *GL1*, which is required for trichome production [64], was also increased in *sk372* in which trichome production was actually suppressed, suggesting that *GL1* was unlikely responsible for the mutant trichome phenotype. Over-expression of Arabidopsis *GL3* alone can result in “hairy canola” with an extremely dense coverage of trichomes on the adaxial leaf surface [40]. In *sk372* glabrous adaxial leaf surface and slight but not significant reduction (*p* > 0.05) of *GL3* and *EGL3* collectively could suggest the possibility that GL3 might be involved in the mutant trichome reduction. However, over-expression of *GL3* in *sk372* only generated single point trichomes in young leaves and was unable to fully recover trichome production as in WT leaves, suggesting that *GL3* alone was not fully responsible for trichome alteration in *sk372*. In this study, *GL2* suppression observed in *sk372* due to the MBW complexes predominantly present in *sk372* might be crucial for the reduced trichome formation and enhanced phenylpropanoid production as found earlier [14]. Moreover, genes coding for the bHLH proteins GL3 and EGL3, GL2, and TTG2 (a WRKY transcription factor) [65] are known being required for trichome production. Possibly significant transcript reduction of *GL2* (*p* < 0.05) together with slight but not significant (*p* > 0.05) reduction of the other three genes (*GL3*, *EGL3*, and *TTG2*) could collectively involve in the trichome phenotypes in *sk372*. In addition, cytokinin (6-benzylaminopurine, BAP) is known being able to induce trichome production in Arabidopsis [66]. Our metabolic profile indicated that in *sk372* glycoside of *trans*-zeatin abundance was increased. Concomitantly the level of active zeatin in *sk372* was likely decreased, which might lead to the suppressed trichome formation in the mutant. Further studies are required to validate these hypotheses. Moreover, the enhanced *TTG1* in *sk372* could be related to the enhanced phenylpropanoids, but unlikely to be responsible for neither the trichome density reduction nor for trichome distortion and altered flavonoid patterns, for *ttg1* mutants typically had few or no trichomes and significantly reduced anthocyanins as reviewed by Broun [11]. It would be interesting to verify the effects of altered expression of the MBW complex components on flavonoid and trichome production in *sk372*.

Moreover, the application of histone deacetylase inhibitor TSA induced the expression of MBW complex genes *MYB75* and *TTG1*, in addition to *TT18* and *TT19*, and slightly suppressed *TT2* expression in the Arabidopsis WT plants (*p* < 0.05), as found similarly in the mutant of *sk372* in which slightly enhanced *TT19* and significantly suppressed *TT2* (p < 0.05) were noted. These findings indicated that histone acetylation is involved in the regulation of the root hair patterning in Arabidopsis seedlings according to Xu et al. [38]. Additionally, the bromodomain protein GTE6 is known to control Arabidopsis leaf development through histone acetylation/deacetylation regulation [67]. Possibility could not be excluded that downregulation of the bromodomain protein gene *BRD5* revealed by microarray analysis in this study might be involved in the epigenetic regulation of the observed phenotypes in *sk372*.

CSN5a is a crucial subunit of the COP9 signalosome (CSN) and removal of RUB1/NEDD8 is performed by CSN5a via its protease activity maintained in JAMMAN domain [20, 33, 34]. CSN5a can also stably exist independent of the CSN in vivo and function actively either as part of the CSN holocomplex or independently [24]. In *sk372*, T-DNA was inserted at the site downstream of JAMM/MPN domain (Fig. 5a), likely maintaining the crucial domain in the truncated CSN5a in *sk372*. Slightly difference in phenotypes in the two Arabidopsis mutants *csn5a-1* (T-DNA inserted immediately downstream of ATG start condon) and *csn5a-2* (T-DNA inserted downstream of JAMM/MPN) were documented by Gusmaroli et al., [20]. It would be interesting to compare the morphological, chemical and transcriptomic changes in *sk372* with those in the above two mutants and try to find out how the remaining domains in the truncated CSN5a in *sk372* affected the function of independent form alone and CSN complexes.

## Conclusions

Defective CSN5a in *sk372* resulted in significant changes in the metabolism of a wide range of phenylpropanoid and carotenoid compounds as well as hormones. These changes in flavonoid and trichome production were likely the consequences of altered transcription of genes associated with the of TTG1/bHLH/MYB complex.

## Additional files

Additional file 1: **Table S1.** Primers used in this study. (XLSX 10 kb)

Additional file 2: **Figure S1.** Overrepresented categories of enriched gene ontology based on differential gene expression between sk372 seedlings compared with wild type Arabidopsis. Circle sizes represent larger or smaller numbers of differentially expressed genes. Circle colours represent p values. (TIF 1781 kb)

Additional file 3: **Table S2.** Down-regulated gene expression more than 2-fold in sk372 compared to wild-type control. (XLSX 25 kb)

Additional file 4: **Table S3.** Up-regulated gene expression more than 2-fold in sk372 compared to wild-type control. (XLSX 31 kb)

Additional file 5: **Figure S2.** Molecular characterization of *sk372*. (A) Southern blot; (B) homozygous determination of sk372. (D) CSN5a truncation analysis using cDNA as a template. Lanes 1 and 2, full length (938 bp) of CSN5a in WT and *sk372*, respectively; Lanes 3 and 4, amplified fragment from 162 bp to 886 bp in WT and *sk372*; Lanes 5 and 6, amplified fragment starting from bp 1034 to 3′ end in WT and *sk372*.The presence of transgene GL3 in the different independent lines of transgenic sk372. M, 1 kb plus DNA marker (Invitrogen); T1-T8, different independent lines of transgenic plants; WT, wild type control. (TIF 7690 kb)

Additional file 6: **Figure S3.** The presence of transgene GL3 in the different independent lines of 563 transgenic sk372. M, 1 kb plus DNA marker (Invitrogen); T1-T8, different independent lines of 564 transgenic plants; WT, wild type control. (TIF 879 kb)

## Plant Materials

Col-4: Columbia ecotype-4 of *Arabidopsis thaliana*
*sk372*: An Arabidopsis T-DNA activation-tagged mutant selected from of the SRC (Saskatoon Research Centre) population
WT: Wild type plants

## Protein complex components and related proteins

bHLH: Basic helix-loop-helix
BRD5: A bromodomain protein
COP9: Constitutive photomorphogenic9
CPC: CAPRICE
CSN5a: COP9 signalosome subunit 5a
EGL3: ENHANCER OF GLABRA3
GL1: GLABRA1
GL2: GLABRA2
GL3: GLABRA3
GPPS: Geranyl diphosphate synthase
JAMM: Jab1/MPN/MOV34 domain
MBW: MYB-bHLH-WDR complexes
MPN: MPR1p and PAD1p amino-terminal
MYB: Myeloblastosis family of transcription factors
TT: Transperant Testa
TTG1: TRANSPARENT TESTA GLABRA1
UGT: UDP-glycosyltransferase

## Chemicals and Bioinformatics and chemical analysis

BHT: Butylated hydroxytoluene
DEGs: Differentially expressed genes
GO: Gene Ontology
HPLC: High-performance liquid chromatography
LC-MS^2^: Liquid chromatography-tandem mass spectrometry
MS: Murashige and Skoog medium for tissue culture
Q-PCR: Real-time quantitative PCR
TSA: Trichostatin A, a histone deacetylation inhibitor
UV: Ultraviolet light
