# Supplementary figures and images for: COP9 signalosome subunit 5A affects phenylpropanoid metabolism, trichome formation and transcription of key genes of a regulatory tri-protein complex in Arabidopsis
Source: BMC Plant Biol. 2018 Jun 25;18:134. doi: 10.1186/s12870-018-1347-9 (PMC6020244; doi:10.1186/s12870-018-1347-9)

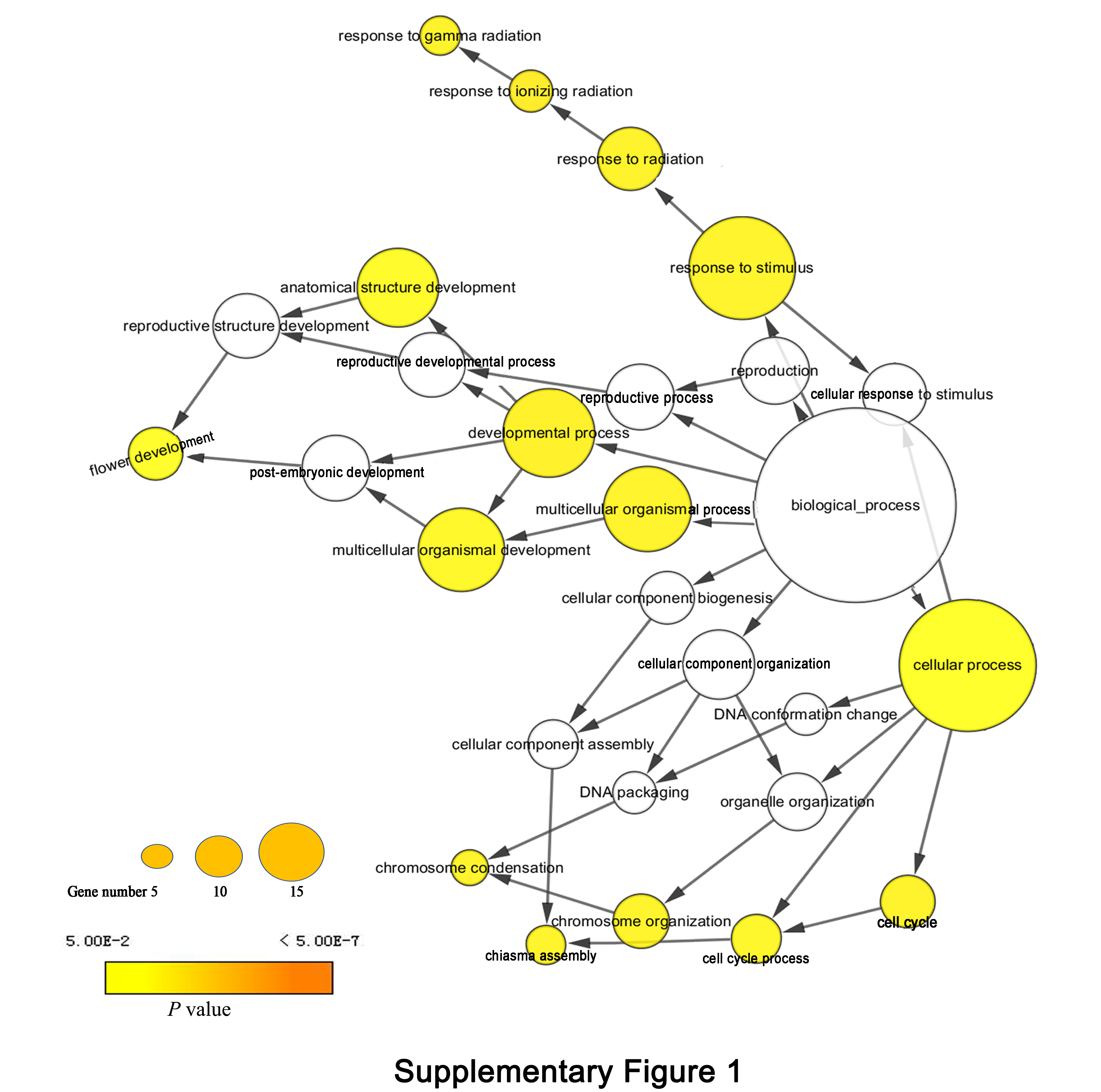

Supplement: Supplementary file 2 — Figure S1. Overrepresented categories of enriched gene ontology based on differential gene expression between sk372 seedlings compared with wild type Arabidopsis. Circle sizes represent larger or smaller numbers of differentially expressed genes. Circle colours represent p values. (TIF 1781 kb) [file 12870_2018_1347_MOESM2_ESM.tif]

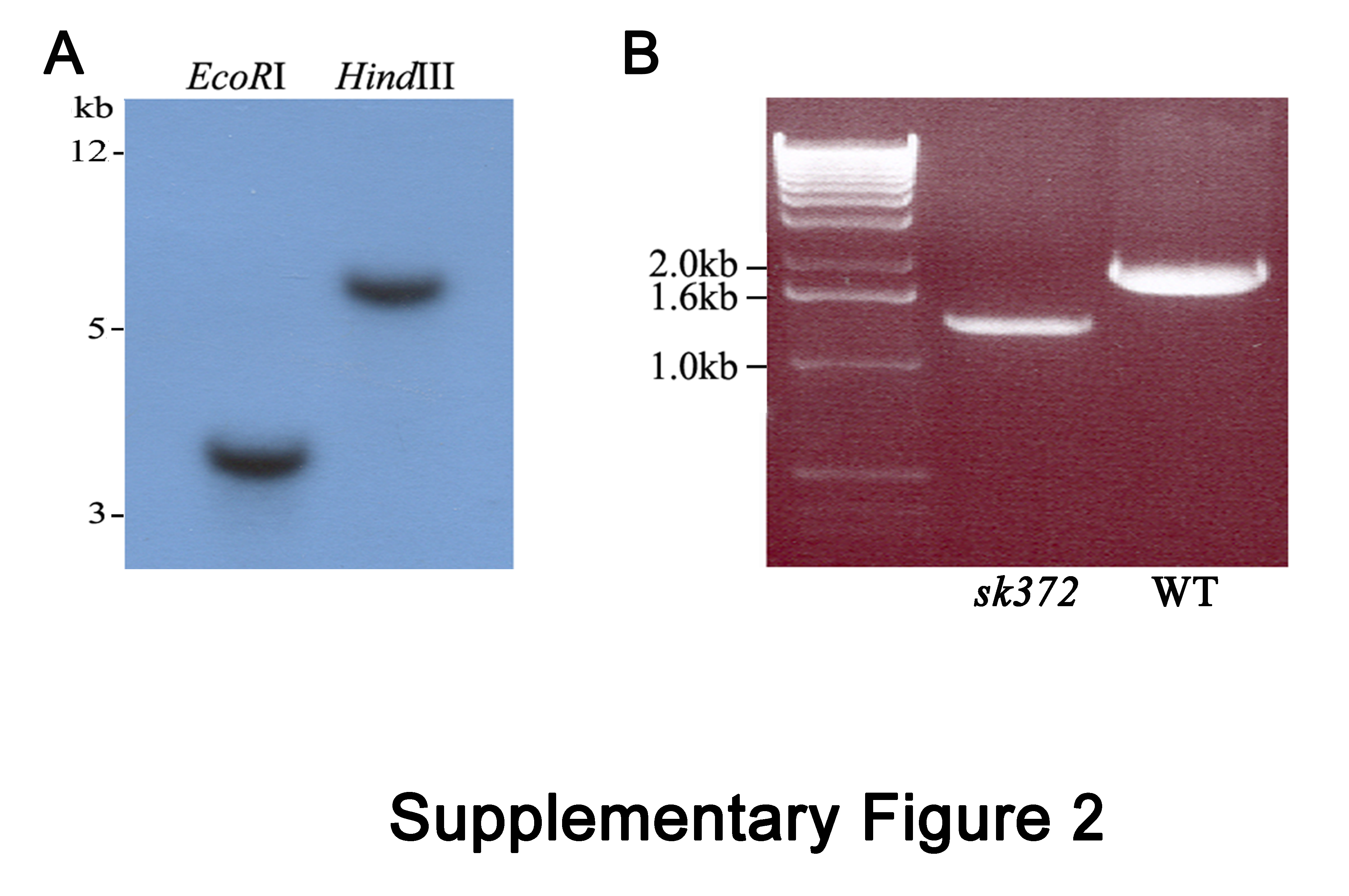

Supplement: Supplementary file 5 — Figure S2. Molecular characterization of sk372. (A) Southern blot; (B) homozygous determination of sk372. (D) CSN5a truncation analysis using cDNA as a template. Lanes 1 and 2, full length (938 bp) of CSN5a in WT and sk372, respectively; Lanes 3 and 4, amplified fragment from 162 bp to 886 bp in WT and sk372; Lanes 5 and 6, amplified fragment starting from bp 1034 to 3′ end in WT and sk372.The presence of transgene GL3 in the different independent lines of transgenic sk372. M, 1 kb plus DNA marker (Invitrogen); T1-T8, different independent lines of transgenic plants; WT, wild type control. (TIF 7690 kb) [file 12870_2018_1347_MOESM5_ESM.tif]

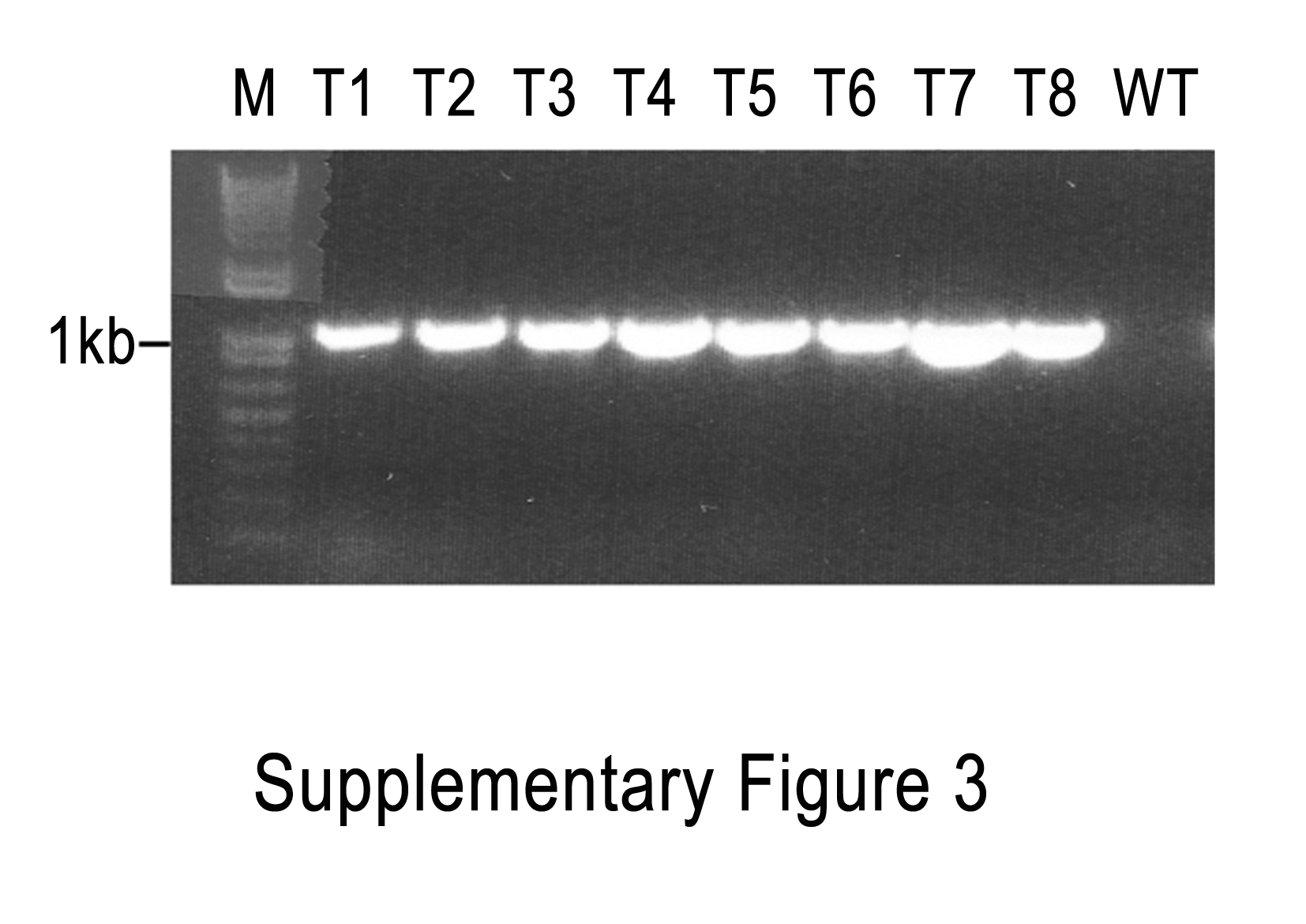

Supplement: Supplementary file 6 — Figure S3. The presence of transgene GL3 in the different independent lines of 563 transgenic sk372. M, 1 kb plus DNA marker (Invitrogen); T1-T8, different independent lines of 564 transgenic plants; WT, wild type control. (TIF 879 kb) [file 12870_2018_1347_MOESM6_ESM.tif]
